# Application of Quantitative Magnetic Resonance Imaging (QMRI) to Evaluate the Effectiveness of Ultrasonic Atomization of Water in Truffle Preservation

**DOI:** 10.3390/jof10100717

**Published:** 2024-10-15

**Authors:** Alessia Marino, Marco Leonardi, Alessandra Zambonelli, Mirco Iotti, Angelo Galante

**Affiliations:** 1 Department of Life, Health and Environmental Sciences (MESVA), University of L’Aquila, Via Vetoio, 67100 L’Aquila, Italy; alessia.marino2@graduate.univaq.it (A.M.); marco.leonardi@univaq.it (M.L.); angelo.galante@univaq.it (A.G.); 2 Department of Agricultural and Food Sciences, University of Bologna, Viale G. Fanin 44, 40127 Bologna, Italy; alessandr.zambonelli@unibo.it; 3 Gran Sasso National Laboratory (LNGS), National Institute for Nuclear Physics (INFN), 67100 L’Aquila, Italy; 4 Department of Physical and Chemical Sciences, CNR-SPIN Institute, 67100 L’Aquila, Italy

**Keywords:** *Tuber borchii*, *Tuber melanosporum*, shelf-life, ascoma mass, water content, ascoma volume, apparent diffusion coefficient, T1 and T2 relaxation times

## Abstract

Truffles of the *Tuber* genus (Pezizales, Ascomycetes) are among the most valuable and expensive foods, but their shelf life is limited to 7–10 days when stored at 4 °C. Alternative preservation methods have been proposed to extend their shelf life, though they may alter certain quality parameters. Recently, a hypogeal display case equipped with an ultrasonic humidity system (HDC) was developed, extending the shelf life to 2–3 weeks, depending on the truffle species. This study assesses the efficacy of HDC in preserving *Tuber melanosporum* and *Tuber borchii* ascomata over 16 days, using quantitative magnetic resonance imaging (QMRI) to monitor water content and other parameters. Sixteen *T. melanosporum* and six *T. borchii* ascomata were stored at 4 °C in an HDC or a static fridge (SF) as controls. QMRI confirmed that *T. borchii* has a shorter shelf life than *T. melanosporum* under all conditions. HDC reduced the rate of shrinkage, water, and mass loss in both species. Additionally, the Apparent Diffusion Coefficient (ADC), longitudinal relaxation time (T1), and transverse relaxation time (T2), which reflect molecular changes, decreased more slowly in HDC than SF. QMRI proves useful for studying water-rich samples and assessing truffle preservation technologies. Further optimization of this method for industrial use is needed.

## 1. Introduction

*Tuber* species (Pezizales, Ascomycetes) form edible hypogeous ascomata (called truffles or true truffles) with a unique and appreciated aroma that makes them one of the most expensive foods in the world [1]. At least 180 species of *Tuber* (Pezizales, Ascomycetes) have been estimated worldwide [2] and a few are sold for hundreds or thousands of euros per kilo. In 2022 the global truffle market size was estimated at USD 583.9 million and it is expected to grow consistently in the next few years (https://www.grandviewresearch.com/industry-analysis/truffle-market-report; accessed on 31 August 2024). Among the *Tuber* species with a black and warty peridium, *Tuber melanosporum* Vittad. is the most valuable and it is widely cultivated in many European and extra-European countries [3,4]. In turn, the market of truffles with pale and smooth peridium is dominated by *Tuber magnatum* Picco although the interest in the whitish truffle *Tuber borchii* Vittad. is increasing worldwide [5,6].

The increasing demand for truffles worldwide has resulted in the development of technologies to improve the preservation and to extend the shelf life of these valuable fungi [1]. Truffles exhibit their best sensorial properties at maturity and then quickly lose their typical taste, firmness, and smell a few days after harvest [7,8,9]. Refrigeration at 4 °C remains the main approach to preserve truffles at the commercial level although many methods such as modified atmosphere packaging, hypobaric packaging, drying, canning, freezing, irradiation, sonication, surface sterilization with chemicals, gelatine, or biofilm deposition have been tested to extend the shelf life of ascomata [9,10,11,12,13,14,15,16,17,18,19,20,21]. However, these additional or alternative treatments may be responsible for changes in one or more of the ascoma quality parameters. Moreover, most of the studies conducted until now evaluated the effects of preservation treatments on the microbial community, on the volatile organic compound composition, and, to a lesser extent, on the nutritional properties of preserved truffles.

Water is the major component of fresh ascomata but, surprisingly, it is scarcely considered to grade the quality and commercial value of truffle. All biotic and biochemical activities as well as some qualitative parameters (e.g., freshness, firmness) depend on the content and dynamics of water within the ascomata. Water content can be easily measured by standard wet chemistry, but this approach compromises the ascoma integrity, does not provide any further information, and prevents the possibility of performing longitudinal studies over time. On the contrary, low-field nuclear magnetic resonance (LF-NMR) and magnetic resonance imaging (MRI) can be used to evaluate both water status and water dynamics in mushrooms without damaging them [22]. Recently, Galante et al. [23] have demonstrated that quantitative magnetic resonance imaging (QMRI) can be effectively applied to truffles for monitoring water content and other parameters related to the water behavior, like its mobility or molecular interactions with the surrounding micro-environment. This technology is non-invasive, cost-effective, time-saving, and suitable for analyzing the same truffle several times over the investigation period, thus making QMRI an interesting option to test storage technologies. At the same time, the reduced cost and the chance to extract several quantitative parameters within short scanning times make QMRI also suitable for industrial applications.

Recently, a new technology consisting of a hypogeal display case equipped with an ultrasound humidity emanation system (HDC) has been specifically developed to preserve fresh truffles (https://www.afoodtartufi.it/it/hypogeal-display-case/; accessed on 31 August 2024). This technology would allow for an increase in the shelf life of fresh ascomata until 2–3 weeks, according to the truffle species. In this study, we tested the efficiency of the ultrasound humidity emanation technology in extending the shelf life of fresh truffles by using QMRI as the analytical technique. We analyzed the variation in a series of QMRI parameters in ascomata of *T. melanosporum* and *T. borchii* stored at 4 °C for 17 days in a static fridge (controls) or in the HDC.

## 2. Materials and Methods

### 2.1. Ascoma Selection and Preparation

The ascomata of *T. melanosporum* and *T. borchii* and the HDC used in this study were provided by Appennino Food company (Savigno, Valsamoggia, Italy, https://www.afood.it/ENG/default.aspx; accessed on 31 August 2024). Ascomata were collected in January 2023 from natural truffle orchards of south Italy (Basilicata and Calabria) for *T. borchii* and central Italy (Abruzzo) for *T. melanosporum*. The exact collection localities are unknown because ascomata were selected from commercial batches of fresh truffles. The ascomata were stored for 4 d at 3 ± 1 °C pending QMRI analyses. Sixteen ascomata of *T. melanosporum* and six ascomata of *T. borchii* were selected following the criteria described by Galante et al. [23], namely, regular shape, integrity of the peridium, no signs of rot or decay, and being size compatible with the inner diameter of the MRI hardware (3.4 cm). Unfortunately, no other *T. borchii* ascomata meeting these criteria were found at the time of the study.

The mass (M_A_) of each selected ascoma was measured with an analytical balance before and after each QMRI round of analysis (Kern EMB 100–3, Merk, Darmstadt, Germany). Half of the ascomata of each truffle species were stored in the HDC, with a level of air humidity of 80% and a temperature of 4 °C. HDC was equipped with the commercial humidifier Limpia 4 (Olimpia Splendid, Cellatica, Italy) with a capacity of 300 mL h^−1^. The other ascomata were preserved in a static refrigerator (4 °C, SF) and were used as controls. After the last QMRI round of measurement, all ascomata were lyophilized by a 2K Benchtop freeze dryer (VirTis SP Scientific, American Laboratory Trading, East Lyme, CT) for 3 days to measure their dry mass. The species identity of each selected ascoma was confirmed at the end of the experiment by direct PCR amplification of dried gleba fragments [24] using the species-specific pairs for *T. borchii* [25] and the black truffles *T. melanosporum* and *Tuber brumale* [26] which are sometimes misidentified by considering only its peridium morphology and aroma. Molecular confirmation of ascoma identity was achieved at the end of the experiment to avoid any alteration of the peridium integrity. Molecular analyses confirmed the identity of all 6 *T. borchii* ascomata selected for QMRI measurements. On the contrary, 2 out of 8 *T. melanosporum* ascomata preserved in the HDC were identified as *T. brumale* and their respective QMRI data were then excluded from the analyses.

### 2.2. QMRI Acquisitions

MRI scans were performed with a preclinical M2^TM^ compact high-performance MRI system (Aspect Imaging, Shoham, Israel) with 1.0 T magnetic field (proton frequency of 45 MHz) equipped with a cylindrical solenoid radiofrequency coil, 8 cm long and 3.5 cm of inner diameter, which limited the size of samples. The first MRI acquisition was performed four and five days after harvesting for *T. borchii* and *T. melanosporum*, respectively. Then, the ascomata underwent MRI acquisition every 2–4 days for a total of six measurement rounds, over a monitoring period of 17 days. The two truffle species were analyzed on different (consecutive) days because the extensive protocol of sequence acquisition used for each ascoma prevented analyzing all the 22 ascomata in a single day. The analytical protocol was planned with the constraint to return all ascomata back to the SF or the HDC within 45 min after picking (30 min for acclimation at room temperature and 14 min for QMRI acquisition), to minimize the impact of each measurement on the preservation state. Acclimatization time is required because some physical parameters measured by QMRI might be temperature dependent. Each ascoma was always repositioned with the same side facing the QMRI sample holder at every measurement round. Even if the ascoma changed size (and partially also shape) during the monitoring period, the care in positioning was devoted to minimizing intra-ascoma fluctuation, offering the same 2D slice for the acquisition. The imaging sequence was based on Spin Echo (SE) to (i) minimize the effects of spatial magnetic field inhomogeneities, a typical condition for low-cost LF-NMR scanners with the main magnetic field generated by permanent magnets, and (ii) maximizing the Signal to Noise Ratio (SNR) of each acquisition. For each session, we employed a slightly modified protocol with respect to Galante et al. [23] with only one average for each scan to reduce the total scanning time. As in Galante et al. [23], five physical parameters were measured for each ascoma: average free water fraction (WF, where free refers to high-mobile water molecules with relaxation times larger than several ms), volume (V), Apparent Diffusion Coefficient (ADC), longitudinal relaxation time (T1), and transverse relaxation time (T2). For T1 and T2, we acquired images of a single central coronal slice, 1 mm thick, 36 × 80 mm Field of View, and 1 × 1 mm in-plane resolution, including a small reference phantom of doped water (1.5 mL tube with 6 mM CuSO_4_ in water). For T1 determination, we used Echo Time TE = 4.9 ms and Repetition Time TR = 50, 150, 300, 600, 1000, 1600 ms. For T2 determination, we used a CPMG acquisition (Car-Purcell–Meiboom–Gill, 128 echos, TE = 4.4 ms). In the latter case, to reduce the TE, we disabled all gradient fields, collecting echoes from the entire ascomata (no water phantom was present) without any spatial information. The protocol included SE acquisitions of the same central slice devoted to the determination of ADC, by using TE = 22.3 ms, TR = 1600 ms, and b = 0, 40.5, 162, and 365 mm^2^ s^−1^. All quantitative parameters (T1, T2, ADC) were calculated on a voxel-by-voxel basis from a series of images with different sequence parameters (TR, TE, and b, respectively): they were the outcome of exponential fits, performed using in-house developed Matlab scripts (The MathWorks, Inc., Natick, MA, USA) based on the Levenberg–Marquardt nonlinear least-squares’ algorithm, according to the standard formulas for the NMR signal dependence with the acquisition sequence parameters [27].

On a voxel-by-voxel analysis, WF is defined as the fraction of the ascoma average Proton Density (PD) and the doped water PD, with both PDs extracted from the T1 fits. It is a dimensionless parameter defined in a 0 to 1 interval, where 0 corresponds to no water and 1 to bulk water. After removing the edge voxels to avoid partial filling effects, we averaged the water fraction on each pixel of the central slice to define the average ascoma’s WF.

Moreover, unlike Galante et al. [23], the V_MRI_ of each ascoma was accurately measured using a multislice SE (i.e., 3D imaging) to image the entire ascomata (0.62 × 0.62 × 1.1 mm^3^ resolution, 0.42 mm^3^ per pixel, TE = 5.5 ms, TR = 1000 ms). To obtain the ascoma volume, we first defined a threshold above the noise level for the ascoma voxel intensity. The threshold was used to identify the connected 3D cluster of pixels with above-threshold intensity as the ascoma. We computed the number of pixels on the cluster’s frontier (N_skin_) as well the total number of cluster’s pixels (N_bulk_). Voxels on the ascoma’s edge can be partially filled: on average we can consider them as half-filled by the ascoma. This translates into volume determination as V = (N_bulk_ − N_skin_/2) ⋅ V_pixel_, where we used the pixels’ volume (V_pixel_) to express the result in mm^3^.

Bound water has much shorter T2 than free water since the water motion is responsible for a time averaging of the local magnetic fields inhomogeneities and thus increased relaxivities. If T2 of bound water is shorter than TE, it is not measurable by our scanner and will not contribute to the measured parameters. In our scans, MRI signal comes mainly from free water which we can expect to have density close to the bulk water one. Galante et al. (2022) demonstrated that most of the water within fresh ascomata is free. So, we can expect to extract the ascoma’s water content weight ($M_{H_{2}O}$) from

$$M_{H_{2}O}=WF\cdot V\cdot \rho_{H_{2}O}$$

where $\rho_{H_{2}O}$ is the standard water density (1 g cm^−3^).

The total ascoma mass (M_A_) can thus be written as the sum of its water content ($M_{H_{2}O}$) and its residual mass (M_residual_), where the latter refers to the mass which gives no observable MRI signal:

$$M_{A}=M_{residual}+M_{H_{2}O}$$

Hence, the residual mass for each time point may be calculated as the difference between M_A_, measured by the analytical balance, and $M_{H_{2}O}$, obtained by QMRI evaluation of WF and V.

At the end of the protocol, M_residual_ evaluated by QMRI was compared with the dry mass measured by the analytical balance after ascoma lyophilization.

### 2.3. Statistical Analysis

The differences between the value of each parameter (M_A_, WF, V, ADC, T1, T2) from the first round of MRI analyses and those obtained in the following rounds were calculated. The Student’s *t*-test was then used to compare the differences between means of each parameter from ascomata stored in SF and HDC. The graphical trends of the M_A_, V, and ADC (except for the ascomata preserved in the SF) with time (t) for each ascoma were deduced by the formula:

$$y\left(t\right)=a\cdot e^{-\frac{t}{b}}+c$$

where a + c represents the value extrapolated at time t = 0 (the day of ascoma’s arrival in the lab), c the asymptotic value to be reached for very large t values, and b is a constant with dimension of time that describes the rapidity of changes. For small b, the considered variable (M_A_, V, or ADC) has a fast approach to its asymptotic value whereas for large *b* the approach is slower. This means that, the changes in the considered variables are slower within an ascoma showing larger b values (i.e., longer shelf life).

## 3. Results

### 3.1. Ascoma Characteristics

On the day of the first round of MRI measurement (4–5 d after harvesting), the mean mass of the ascomata was 11.51 ± 1.69 g and 7.23 ± 1.25 g for *T. borchii* and *T. melanosporum*, respectively (Table 1). Ascomata selected for preservation in the SF or HDC did not differ significantly in M_A_, V, and WF at the beginning of the study period. An inspection of ascomata after the last session of QMRI measurement did not show evidence of rot and larval cavities in the gleba and peridium. After lyophilization, the dry mass measured by the analytical balance was 3.26 ± 0.58 g and 2.56 ± 0.49 g for *T. borchii* and *T. melanosporum*, respectively (Table 1).

### 3.2. Ascoma Volume (V)

**Figure 1:**
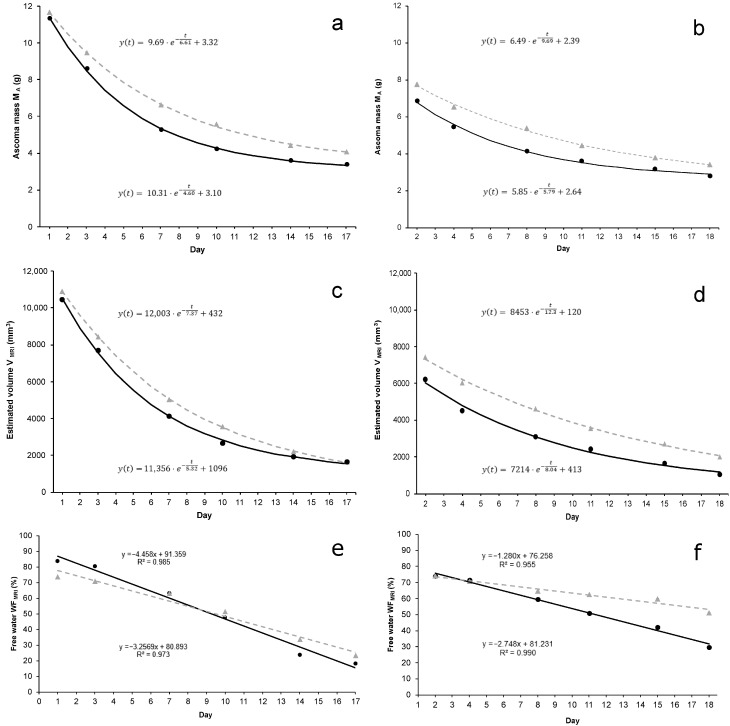
Variation in ascoma parameters and associated trend lines throughout the period of MRI investigation. (**a**) ascoma mass of *T. borchii*; (**b**) ascoma mass of *T. melanosporum*; (**c**) MRI-estimated volume of *T. borchii* ascomata; (**d**) MRI-estimated volume of *T. melanosporum* ascomata; (**e**) free water fraction of *T. borchii* ascomata; (**f**) free water fraction of *T. melanosporum* ascomata. Data from ascomata preserved in the hypogeal display case (HDC) and the static fridge (SF) are visualized in grey (triangles and dotted line) and black (circles and solid line), respectively.

**Figure 2:**
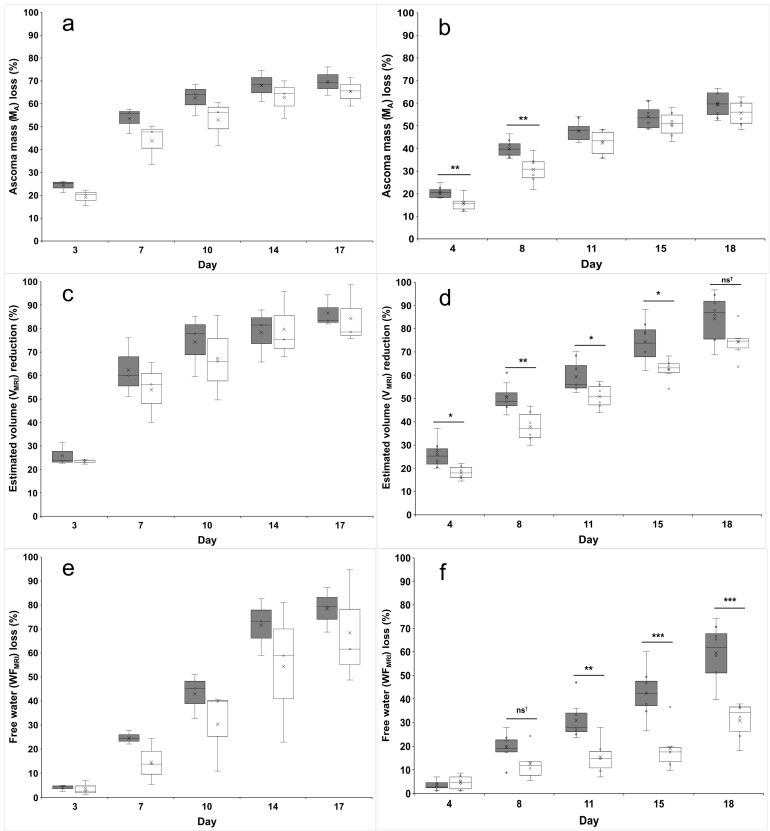
Comparison of ascoma mass (**a**,**b**), MRI-estimated volume (**c**,**d**) and free water fraction (**e**,**f**) percentage reduction between ascomata stored in the static fridge (SF, gray boxes) and the hypogeal display case (HDC, white boxes). (**a**,**c**,**e**) *T. borchii* ascomata; (**b**,**d**,**f**) *T. melanosporum* ascomata. Percentage reductions and statistics were calculated on the differences between values obtained in the first MRI round of measurement and those obtained in the following rounds. Symbols: *p* < 0.06; * *p* < 0.05; ** *p* < 0.01; *** *p* < 0.001; ns^†^ *p* < 0.07.

Sixteen days after the first MRI measurement, V decreased by 86% (from 29.5 ± 4.8 to 4.2 ± 2.8 cm^3^) and 79% (from 18.5 ± 3.5 to 3.9 ± 2.0 cm^3^) for *T. borchii* and *T. melanosporum*, respectively (Figure 1b,c). As for the ascoma mass, volume decreased exponentially regardless of *Tuber* species and preservation method. When compared with the first QMRI measurement round, the reduction in the volume was always higher for ascomata preserved in SF, although significant differences were only found for *T. melanosporum* (Figure 2c,d). Also, the means of the time constant *b* of the curve fitting were higher for ascomata preserved in the HDC than in the SF although no significant differences were found.

### 3.3. Ascoma Mass (M_A_)

M_A_ during the 17 days of investigation decreased exponentially regardless of the *Tuber* species and the preservation method. *T. borchii* and *T. melanosporum* ascomata lost 67% and 58% of their initial M_A_, respectively. The average M_A_ value has always remained higher for both *T. borchii* and *T. melanosporum* ascomata preserved in the HDC throughout all QMRI measurements (Figure 1a,b). This behaviour was confirmed by the time constant *b* of the curve fitting, although a significant difference in this parameter between the two preservation methods was only found for *T. melanosporum* (*p* = 0.02). In particular, the percentage M_A_ loss between SF and HDC was higher in the first half of the study period for both truffle species (Figure 2a,b).

### 3.4. Free-Water Fraction (WF) and Residual Mass (M_residual_)

WF decreased linearly throughout the 17 days of QMRI investigation by 79% to 21% and from 74% to 39% for *T. borchii* and *T. melanosporum* ascomata, respectively. Water loss was faster in *T. borchii* than *T. melanosporum* ascomata (Figure 1e,f). Marked differences were found between ascomata preserved in HDC and SF and the gap between their mean values increased progressively during the study period. In particular, highly significant water losses occurred in *T. melanosporum* ascomata preserved in SF from the 10th day after the first QMRI measurement (Figure 2e,f).

M_residual_ estimated by using V and WF data from QMRI measurements was mostly lower than the dry mass of the respective ascoma measured after lyophilization (Figure S1).

### 3.5. ADC, T1, and T2

ADC, T1, and T2 values have decreased throughout the study period with different trends depending on the truffle species and the method of preservation. No significant differences between ascomata preserved in HDC or SF were found for any parameter at the beginning of the study period.

During the preservation, the MRI signal was reduced along with the ascoma water content. It turns out that all the measurements were characterized by decreasing SNR with time. This effect was more evident for ADC, which adopts a longer echo time and codifies water diffusion in an extra signal reduction compared with the T1 and T2 measurements (i.e., images used to extract ADC data have lower SNR). With the adopted protocol, the SNR for ADC measurements became too low to produce reliable results by the sixth round of QMRI measurement and, for this reason, ADC measurements are provided for five rather than six rounds of QMRI measurements.

The ADC showed a similar exponentially decreasing trend for all ascomata except those of *T. borchii* preserved in the SF (Figure S2a,b). For these latter ascomata, ADC remained broadly stable between the first two rounds of MRI measurement and then decreased more rapidly in the remaining period of preservation. No significant differences between ascomata preserved in SF and HDC were found either for *T. melanosporum* nor for *T. borchii* (Figure S3a,b). However, *T. melanosporum* ascomata from HDC showed a slower decline in the ADC, particularly halfway through the study period (*p* = 0.057 on the 11th day).

Changes in T1 and T2 relaxation times differed between the truffle species. T1 decreased rather linearly throughout the period of investigation for *T. borchii* whereas two phases with different slopes were visible for *T. melanosporum*: the first with a smaller slope until the third QMRI measurement round and the second with a higher slope starting from the fourth round (Figure S2c,d). Regardless of the truffle species, the T1 of ascomata preserved in the HDC decreased slower as early as the second round of QMRI measurement for *T. borchii* and the fourth round for *T. melanosporum*. No statistical significances were found for this parameter although a couple of almost significant *p* values were found for ascomata of *T. borchii* (*p* = 0.054 on the 3rd day) or *T. melanosporum* (*p* = 0.073 on the 14th day) preserved in the HDC (Figure S3c,d). The T2 relaxation time decreased more quickly for *T. borchii* than *T. melanosporum* and for ascomata preserved in the SF than HDC (Figure S2e,f), but no significant differences were found between the two preservation methods (Figure S3e,f).

## 4. Discussion

The ultrasonic atomization of water is a technology with a wide range of applications in household air humidification, water desalination [28], humidification of fuel cells [29], ethanol–water separation, and medical devices [30]. An ultrasonic humidifier coupled with cold storage is a suited technology in preserving the quality of fruit and vegetables and in reducing their water losses [31,32]. The fine mist created by the ultrasonic transducer preserves the product quality by reducing its water and mass losses and increasing its shelf life [32]. For mushrooms, ultrasonication has proven effective in enhancing several processes such as dehydration, the extraction of molecules, freezing and thawing, and frying [33], but it has been also used to improve the humidification system of storage rooms [34]. In this work we evaluated for the first time the efficiency of the ultrasonic atomization of water coupled with cold storage in preserving truffles by acquiring a series of QMRI parameters during 2–3 weeks of preservation. In addition to the content of free water (WF) and the ascoma volume (V), we measured three other physical parameters (ADC, T1, and T2) that are closely related to the changes occurring in the micro-environment of ascomata during their preservation.

WF, M_A_, and V of both *T. borchii* and *T. melanosporum* ascomata decreased with the same trend as previously found for *T. aestivum* by Galante et al. [23]. In our study, the preservation method did not affect the decreasing trend of these three parameters. When comparing the data of the two considered truffle species, the decrease in WF, M_A_, and V seem to be much faster in *T. borchii* than *T. melanosporum*. It is well known that black truffles have a longer shelf-life than white or whitish truffles [35] but the differences in water loss are not due to the thickness or anatomy of the outer cell layers. In fact, *T. borchii* and *T. melanosporum* have both pseudoparenchymatous exoperidia (100–300 µm and 70–150 µm thick, respectively) [36] and, in addition, the presence of warts in *T. melanosporum* increases the air-exposed surface for the same ascoma volume. Furthermore, *T. aestivum* ascomata have a 100–180 µm thick pseudoparenchymatous exoperidium [36] and larger warts, but the water loss rate is about half that of *T. melanosporum* and a third of *T. borchii* stored in the same static fridge [23]. These differences could be attributed to the architecture of the exoperidium cells and/or their chemical composition. In black truffles, cell walls of the outer layers are full of melanin [37], a hygroscopic polymer synthetized by many fungal species in response to a number of environmental stresses including protection against desiccation [38,39]. Moreover, exoperidium cells of *T. melanosporum* and *T. aestivum* have thickened walls with reduced or absent cellular lumen. These differences could also be due to the number and organization of peridium pores which constitute the aeration system of ascomata [40].

Evident differences in WF, M_A_, and V were found between ascomata preserved SF and HDC although statistical significances were only found for *T. melanosporum*. Most probably, the low number of *T. borchii* replicates available for analysis (three replicates per treatment vs. six–eight for *T. melanosporum*) did not allow for the adequate detection of significant differences. In general, truffles of both species stored in HDC experienced a slower decrease in these three parameters than those preserved in the SF. As for *T. aestivum*, the losses of mass and volume are mostly due to the reduction of WF contained in the ascoma.

The differences between M_residual_ derived by QMRI parameters (V and WF), and the mass of lyophilized ascomata could be due to two systematic errors. The first involves the bound water that is invisible to our MRI protocol and introduces a systematic underestimation of WF and hence an overestimation of M_residual_. However, we have indication that this water fraction is negligible with respect to the total amount of water within a truffle [23], and we expect it not to be the case of our study.

The second systematic error could be related to the ascoma’s average WF calculation performed on a single 2D central slice of the ascoma. If the free water is not evenly distributed within the gleba (i.e., larger in the central region and smaller towards the peridium), the average WF value of the ascoma may have been overestimated because the incidence of peripheric voxels (those with lower free water content) was lower in a 2D average than in a full 3D one. The overestimation of the average WF determines an overestimation of $M_{H_{2}O}$, and then an underestimation of M_residual_. This hypothesis was supported by the inspection of images reporting the voxel-by-voxel WF computed from MRI data of the 2D central slices: some ascomata showed a uniform distribution of WF in the entire gleba whereas other ascomata were characterized by a drop of WF from the central to the peripherical voxels. In the latter case, we estimated M_residual_ may have been underestimated by up to 10–20%.

ADC, T1, and T2 parameters of *T. borchii* and *T. melanosporum* ascomata decreased throughout all the period of QMRI investigation and no significant differences were found between the two preservation conditions as found by Galante et al. [23] for *T. aestivum*. However, in this study, we found different behaviours in the decay of these parameters affected both by the truffle species and the method of preservation. These discrepancies could be due to the specific ascoma anatomy of the single species but also to the period of harvesting (February rather than June).

ADC is related to the water mobility and its reduction can indicate the shrinking of the inner porous structure of ascomata (formed by non-water-permeable membranes) following the loss of water and volume but with a mechanism that maintains the integrity of the porous structure itself. On the other hand, an ADC increase can be related to the fainting of such porous structure with a resulting increase in free water mobility. As for WF, MA, and V, the HDC seems to slow the ADC time decrease in its water mobility although the effect on this latter parameter is less evident.

T1 and T2 relaxation times change depending on the molecular environment that the water molecules explore during their random walk. The relaxation times provide valuable information on the molecular environment characterizing a complex biological system like a truffle, although T1 and T2 do not reveal the presence and the role of specific metabolites. Their change in time is an indication of water environment modification, which depends on the biochemical activities that occur during the shelf life of the ascomata. The qualitative picture for these parameters showed that HDC slows down the ascoma changes over time. This could happen by delaying the changes in microbial community during the maturation of truffles [41,42,43], and further studies including different approaches (biochemical and microbial) could help in clarifying this point.

## 5. Conclusions

The ultrasonic humidifier coupled with cold storage has proven to be a viable alternative for the preservation of fresh truffles and it could be easily and cheaply applied on an industrial scale. By increasing the shelf life of both black and white truffles, significant economic losses during the preservation of such valuable foods may be avoided. Weight, volume, andwater content dynamics provided a consistent picture of a slower decrease in time for ascomata stored in HDC. This finding is corroborated by the information provided by the other QMRI parameters (T1, T2, and ADC). They show that the HDC effect is not limited to a slower water loss but also reduces the pace of biochemistry processes that naturally occur during the ascoma’s shelf time. Within our approach, we cannot identify such processes, but we have hints of how an improved preservation method helps to not only retain the water and weight of the fresh ascomata longer but, presumably, also the desired qualitative features. However, the effectiveness of HDC technology should be further evaluated with bigger ascomata (> 3 cm in diameter) because the water behavior could change with the increase in truffle size due to the smaller surface to volume ratio. Once again this shows how QMRI, a non-invasive technique based on the direct detection of water properties, is a useful tool for the study of water-rich samples. This technique is complex, but the current trend is towards the development of easy to use and low-field/low-cost scanners which makes it much more accessible to the research as well as the commercial community.

## Figures and Tables

**Table 1:** Ascoma mass (M_A_), volume (V), free water fraction (WF) of each ascoma measured in the first QMRI round and residual mass calculated by QMRI data (M_residual_) and measured by the analytical balance after lyophilization (M_W_).

Ascoma	Preservation Method ^†^	1st Round of MRI Measurement			Residual Mass	
		M_A_ (g)	V (mm^3^)	WF (%)	M_residual_ (g) ± SD *	(M_W_, g)
Tbo1	SF	11.90	10,520	83	3.29 ± 0.21	3.90
Tbo2	SF	13.02	12,319	86	2.67 ± 0.47	2.82
Tbo3	SF	9.13	8467	82	2.42 ± 0.20	2.65
Tbo4	HDC	13.30	12,457	74	3.89 ± 0.23	4.04
Tbo5	HDC	11.87	11,365	74	3.57 ± 0.15	3.02
Tbo6	HDC	9.83	8923	73	3.01 ± 0.19	3.10
Tme1	SF	4.99	5050	68	1.58 ± 0.10	1.56
Tme2	SF	8.79	7707	74	3.16 ± 0.16	3.71
Tme3	SF	7.14	6623	68	2.46 ± 0.15	2.74
Tme4	SF	6.52	6048	75	2.21 ± 0.20	2.26
Tme5	SF	6.31	5636	72	2.34 ± 0.15	2.61
Tme6	SF	6.15	5735	75	2.12 ± 0.29	2.10
Tme7	SF	6.78	5739	75	2.48 ± 0.22	2.61
Tme8	SF	8.12	7041	85	2.33 ± 0.24	2.35
Tme9	HDC	9.04	7930	82	2.55 ± 0.33	2.44
Tme10	HDC	8.22	8557	72	2.45 ± 0.32	3.06
Tme11	HDC	6.64	5949	74	2.12 ± 0.08	2.84
Tme12	HDC	5.80	5068	75	1.96 ± 0.05	2.45
Tme13	HDC	8.49	8563	68	2.50 ± 0.22	2.62
Tme15	HDC	8.23	8351	74	2.08 ± 0.10	2.50

^†^ = static fridge (SF), hypogeal display case (HDC); * n = 6 (number of MRI rounds).

## Data Availability

The original contributions presented in the study are included in the article/Supplementary Material, further inquiries can be directed to the corresponding authors.

## Supplementary Materials

The following supporting information can be downloaded at: https://www.mdpi.com/article/10.3390/jof10100717/s1, Figure S1: Comparison between the mean residual mass obtained by the data from six rounds of MRI measurements (black triangles with standard deviation) and the weight of the lyophilized ascomata (white circles). (a) *T. borchii* (6 ascomata); (b) *T. melanosporum* (14 ascomata). ST = static fridge; HDC = hypogeal display case. Figure S2: Variation in MRI parameters of *T. borchii* (a,c,e) and *T. melanosporum* (b,d,f) ascomata throughout the period of MRI investigation: (a,b) ADC; (c,d) T1; (e,f) T2. Data from ascomata preserved in the hypogeal display case (HDC) and the static fridge (SF) are visualized in grey (triangles and dotted line) and black (circles and solid line), respectively. Figure S3: Comparison of ADC (a,b), T1 (c,d), and T2 (e,f) percentage reduction between ascomata stored in the static (SF, grey boxes) and the hypogeal display case (HDC, white boxes). (a,c,e) *T. borchii* ascomata; (b,d,f) *T. melanosporum* ascomata. Percentage reductions and statistics were calculated on the differences between values obtained in the first MRI round of measurement and those obtained in the following rounds.
