# Supplementary figures and images for: Application of Quantitative Magnetic Resonance Imaging (QMRI) to Evaluate the Effectiveness of Ultrasonic Atomization of Water in Truffle Preservation
Source: J Fungi (Basel). 2024 Oct 15;10(10):717. doi: 10.3390/jof10100717 (PMC11509026; doi:10.3390/jof10100717)

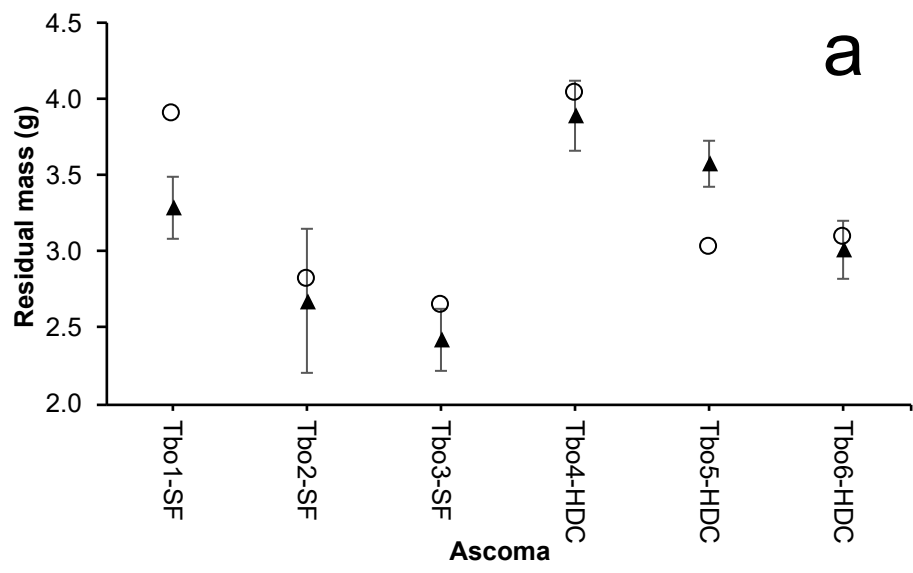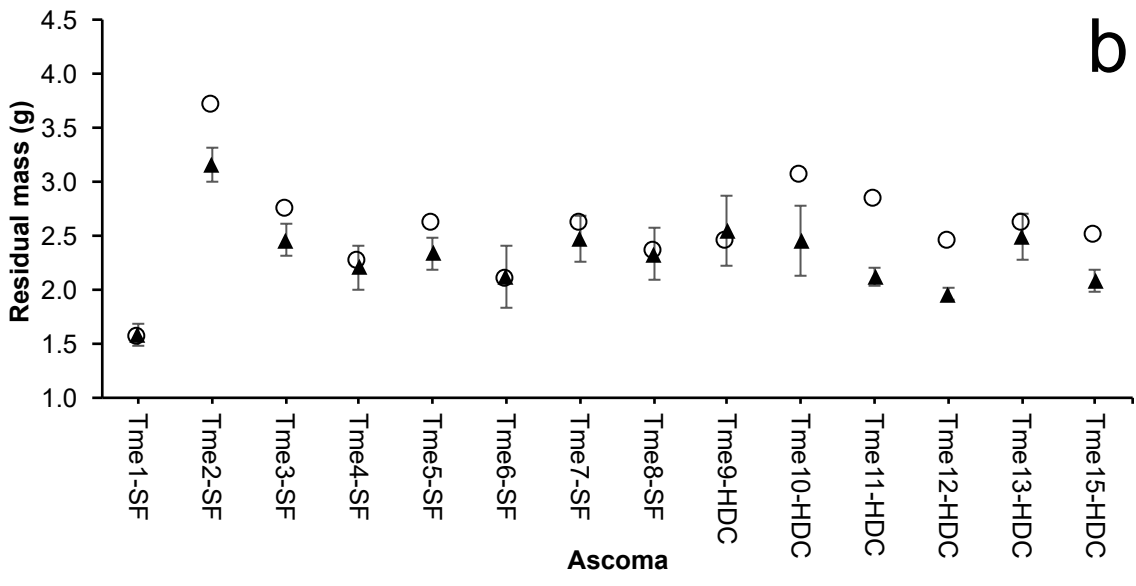

Supplement: Supplementary file 1 [file jof-10-00717-s001.zip › Fig. S1a-b.pdf]

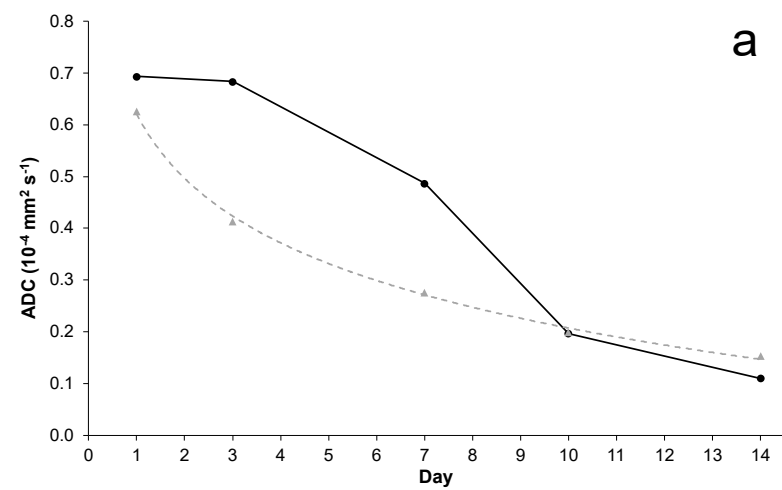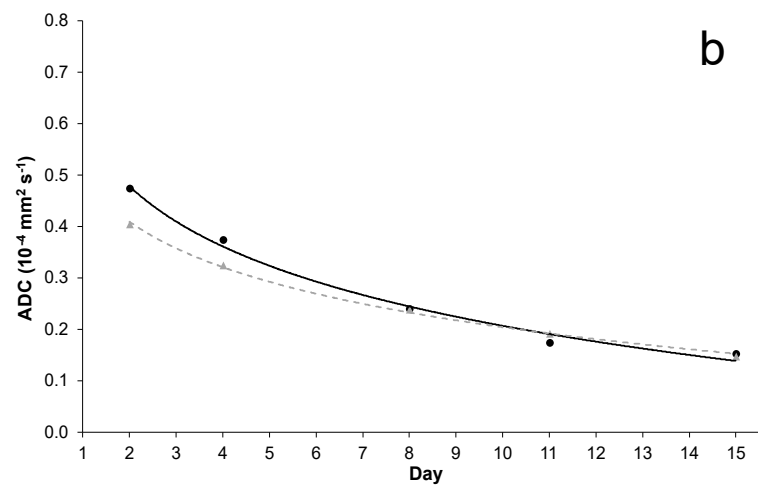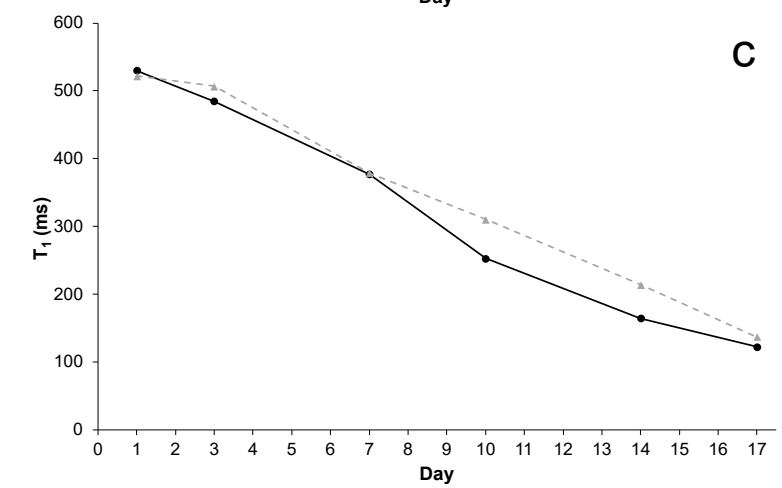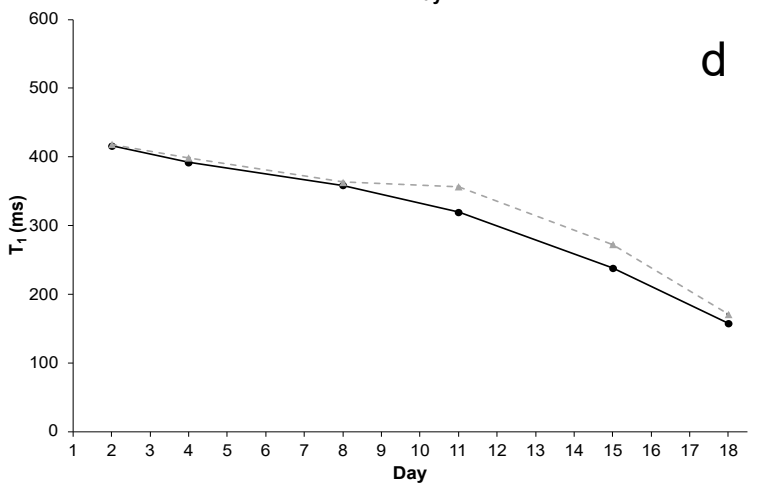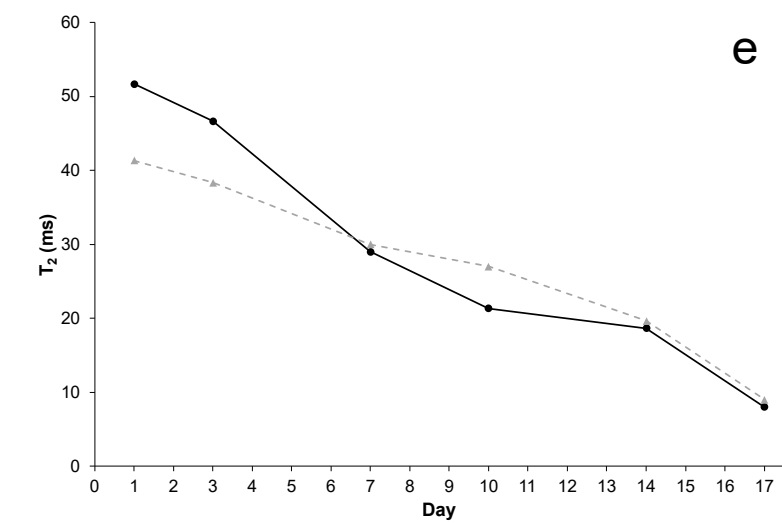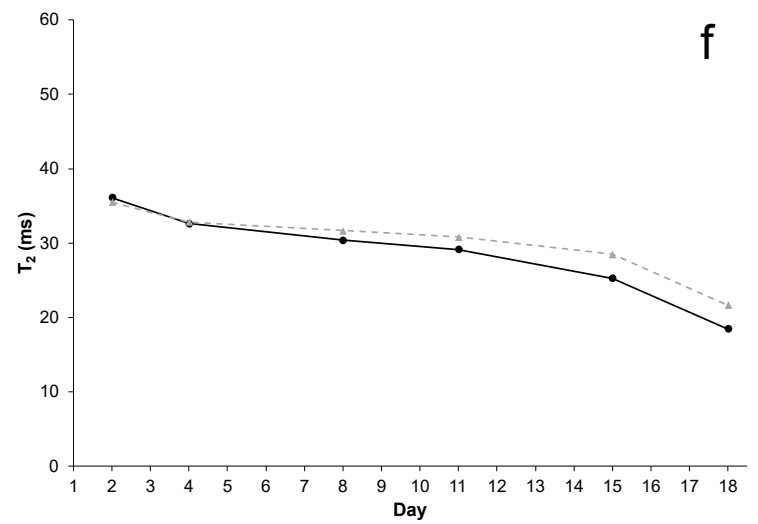

Supplement: Supplementary file 1 [file jof-10-00717-s001.zip › Fig. S2a-f.pdf]

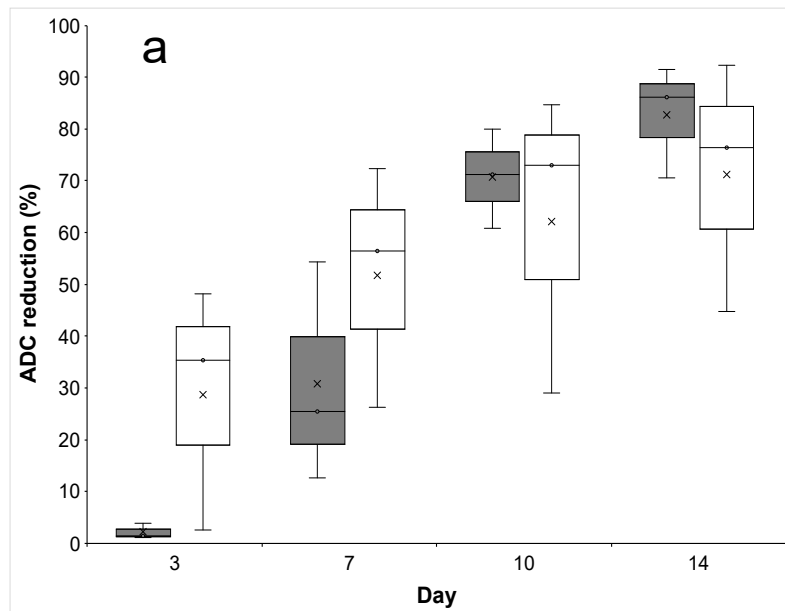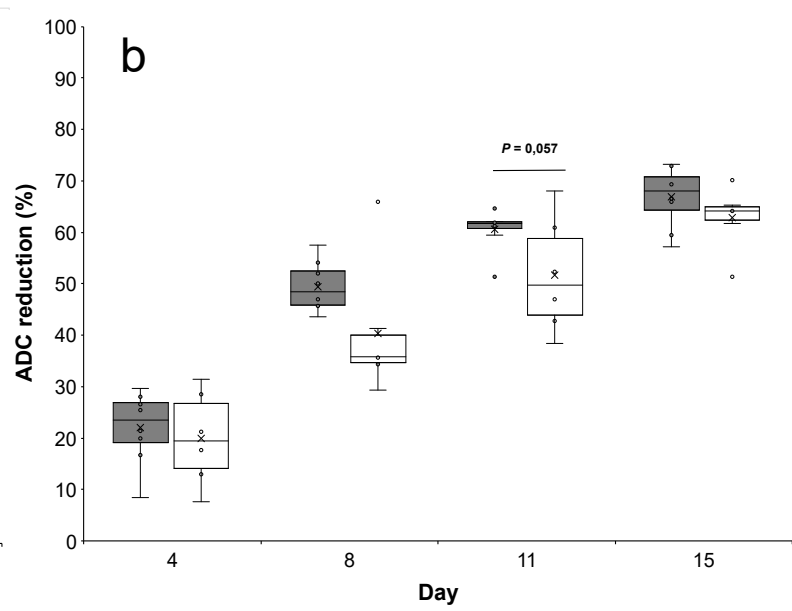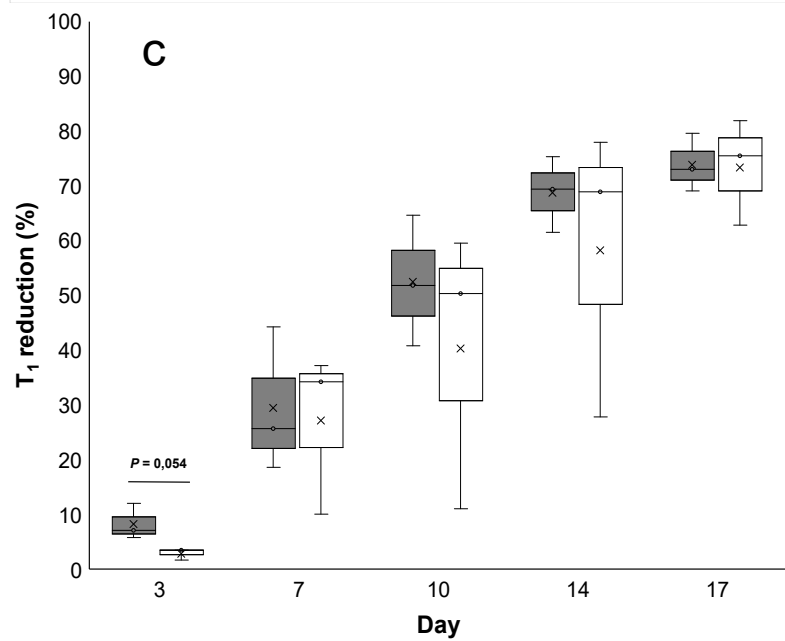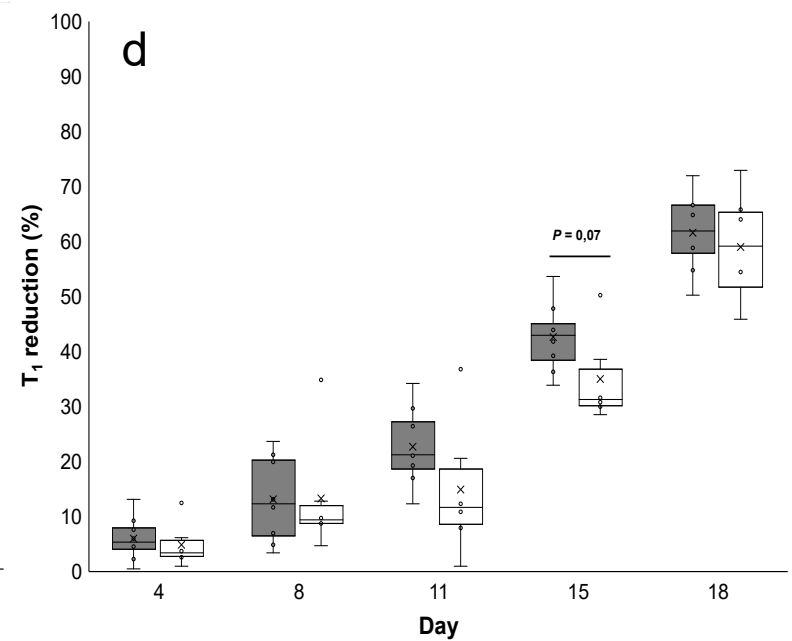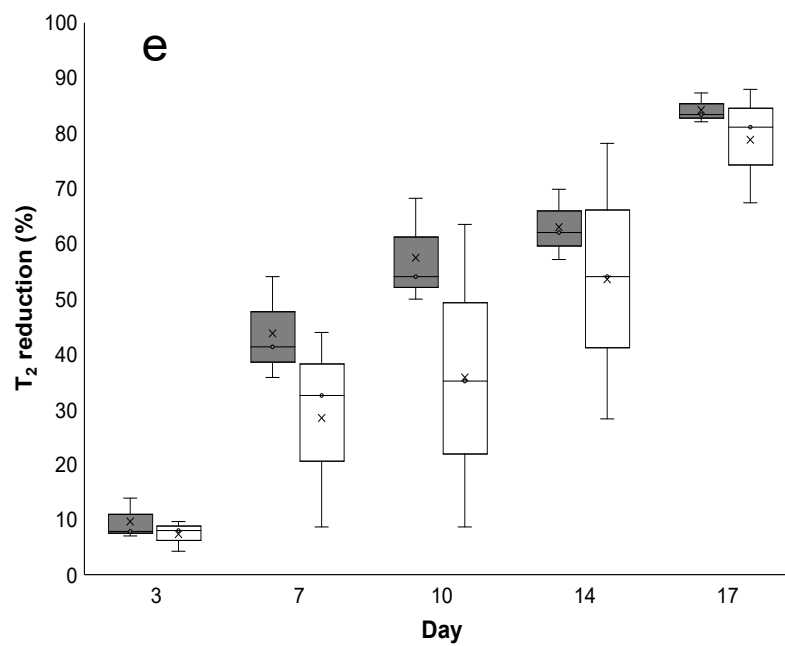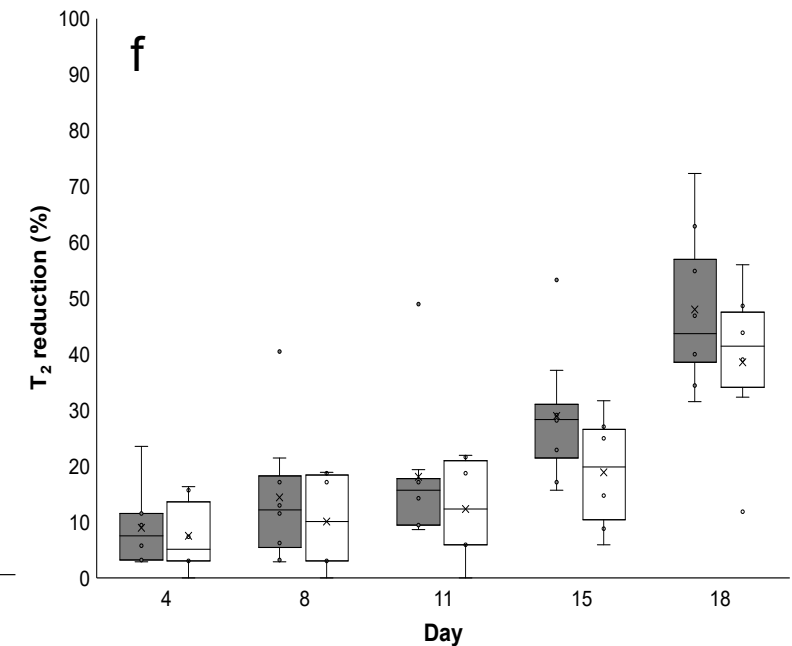

Supplement: Supplementary file 1 [file jof-10-00717-s001.zip › Fig. S3a-f.pdf]
